# Quantitative Mass Density Image Reconstructed from the Complex X-Ray Refractive Index

**DOI:** 10.1371/journal.pone.0131401

**Published:** 2015-06-26

**Authors:** Taihei Mukaide, Atsuo Iida, Masatoshi Watanabe, Kazuhiro Takada, Takashi Noma

**Affiliations:** 1 Nanomaterials R&D Center, Canon Inc., Ohta-ku, Tokyo, Japan; 2 Photon Factory, Institute of Materials Structure Science, High Energy Accelerator Research Organization, Tsukuba, Ibaraki, Japan; University of Nebraska Medical Center, UNITED STATES

## Abstract

We demonstrate a new analytical X-ray computed tomography technique for visualizing and quantifying the mass density of materials comprised of low atomic number elements with unknown atomic ratios. The mass density was obtained from the experimentally observed ratio of the imaginary and real parts of the complex X-ray refractive index. An empirical linear relationship between the X-ray mass attenuation coefficient of the materials and X-ray energy was found for X-ray energies between 8 keV and 30 keV. The mass density image of two polymer fibers was quantified using the proposed technique using a scanning-type X-ray microbeam computed tomography system equipped with a wedge absorber. The reconstructed mass density agrees well with the calculated one.

## Introduction

X-ray imaging is used in a wide variety of applications, from nondestructive testing to medical diagnosis. X-ray imaging techniques are categorized as either absorption or phase contrast imaging [1, 2]. In comparison with absorption contrast imaging, the phase contrast imaging is particularly effective for observing low X-ray absorption materials, such as thin organic materials. Many techniques for X-ray phase contrast imaging have been developed, including a propagation-based method [3], diffraction enhanced imaging [4, 5], grating-based imaging [6, 7], and coherent diffraction imaging [8, 9].

For quantitative materials characterization, it would be beneficial if X-ray images could be used not only to visualize objects but also to quantify the physical or chemical properties of the materials. Quantitative information about structural or physical properties, such as mass density, could be used as simulation input parameters and lead to more reliable simulation results. There is particular interest in developing reliable nondestructive quantification methods to replace destructive methods for the measurement of mass density of organic materials, which is typically measured using ultracentrifuge [10].

For conventional two-dimensional absorption and phase contrast images, quantification of the material properties is difficult because the X-ray path length in the material is usually unknown. For X-ray computed tomography, the electron density is obtained from X-ray phase contrast images [11] because the calculated parameters at each pixel in the reconstructed tomographic image are independent of the X-ray path length. The mass density can be extracted from the electron density of materials with a known chemical composition [12] or, in the case of an unknown chemical composition, by assuming the ratio of the average atomic weight to the atomic number [10, 13]. However, this assumption is not reliable for materials having a large hydrogen content [12].

In this study, a new analytical technique for X-ray computed tomography is proposed. This technique allows visualization of the mass density distribution for low atomic number (*Z*) materials with an unknown composition ratio.

## Materials and Methods

To obtain the mass density, both the real and imaginary parts of the complex X-ray refractive index, which are measured using absorption and phase contrast computed tomography, respectively, are used.

X-ray absorption of a material follows the Beer-Lambert law:

$$\begin{array}{c} I=I_{0}e^{-\mu t}, \end{array}$$ (1)

where *I* and *I*_0_ are the intensity of the transmitted and incident X-rays, respectively, *μ* is the linear attenuation coefficient of the material, and *t* is the thickness of the material. The linear attenuation coefficient of the material is defined using the imaginary part of the complex refractive index (*n* = 1 − *δ* − *iβ*) as

$$\begin{array}{c} \mu =\frac{4\pi }{\lambda }\beta =\rho N_{a}\frac{\sigma_{a}}{\left\langle A\right\rangle }, \end{array}$$ (2)

where *λ* is the wavelength of the X-rays, *ρ* is the mass density of the material, *N*_*a*_ is Avogadro’s number, *σ*_*a*_ is the total atomic cross section per atom, and ⟨*A*⟩ is the average atomic weight of the material.

The X-ray phase shift *ϕ* is defined using the real part of the complex refractive index as

$$\begin{array}{c} \phi =\frac{2\pi }{\lambda }\delta t=\rho r_{0}\lambda tN_{a}\frac{\left\langle Z+f^{\prime }\right\rangle }{\left\langle A\right\rangle }=r_{0}\lambda t\rho_{e}, \end{array}$$ (3)

where *r*_0_ is the classical electron radius, ⟨*Z* + *f*′⟩ is the average of the sum of *Z* and the real part of the anomalous atomic scattering factor (*f*′) for the material, and *ρ*_*e*_ is the electron density of the material. We note that, although the value of *f*′ depends on X-ray energy, it is typically much smaller than the value of *Z* for light elements.

In X-ray computed tomography, the *δ* and *β* images are reconstructed from the projection images of the phase shift and transmittance of X-rays, respectively. The *ρ*_*e*_ distribution is calculated from a reconstructed *δ* tomographic image using Eq (3). Conventionally, the mass density is calculated by assuming that ⟨*Z* + *f*′⟩/⟨*A*⟩ is equal to 0.5 [14] for materials that are made up of light elements. In the case of hydrogen, however, the value of (*Z* + *f*′)/*A* is 0.99. Therefore, using the conventional approximation results in a large error in the calculated mass density of materials having a large hydrogen content, such as polymers and biomolecules. Therefore, there is no practical method for quantitatively evaluating the mass density image of organic materials having a large hydrogen content and an unknown chemical composition.

The proposed analytical procedure for obtaining the mass density uses the both the *δ* and *β* tomographic images. When the chemical composition of the material is known, it is possible to calculate all of the parameters that appear in Eqs (2) and (3) except *ρ*. Based on analysis of a variety of low-*Z* materials, we found that *μ*/*ρ* and *β*/*δ* have a simple empirical relationship. The value of *β*/*δ* is derived from Eqs (2) and (3) as

$$\begin{array}{c} \frac{\beta }{\delta }=\frac{1}{2r_{0}\lambda }\frac{\sigma_{a}}{\left\langle Z+f^{\prime }\right\rangle }. \end{array}$$ (4)

The *μ*/*ρ* image can be calculated from the experimentally obtained *β*/*δ* image. Because the *μ* image can be obtained experimentally directly from absorption tomography, the *ρ* image can be extracted by comparing the calculated *μ*/*ρ* and experimental *μ* images.

**Fig 1:**
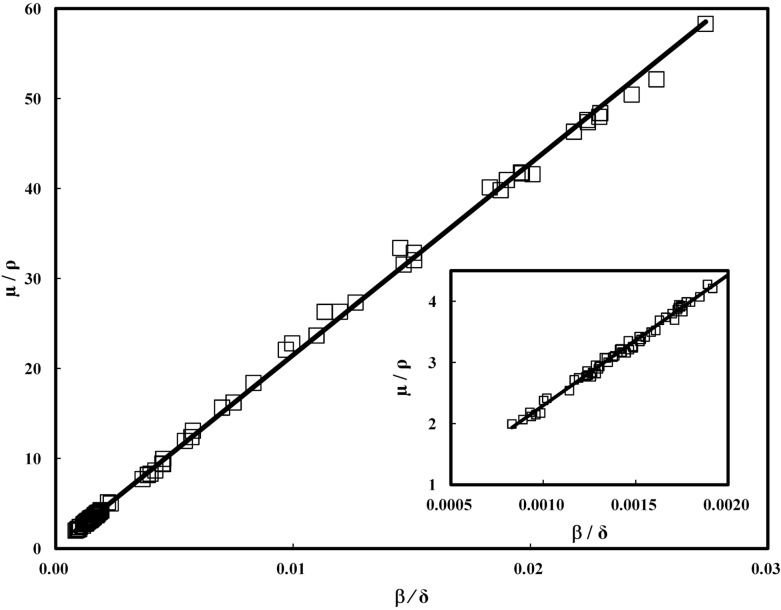
Relationship between *β*/*δ* and *μ*/*ρ*. Calculated relationship between *β*/*δ* and *μ*/*ρ* for 100 materials comprised H, C, N, O, Na, Mg, P, S, Cl, K, and Ca using 10 keV X-rays. The insert shows the magnified figure at *μ*/*ρ* ≤ 0.002. The solid line is the line of best fit by weighted least-squares method. The r-squared value was observed to be 0.998.

Fig 1 shows the calculated relationship between the mass attenuation coefficient (*μ*/*ρ*) and *β*/*δ* for 100 typical materials comprising H, C, N, O, Na, Mg, P, S, Cl, K, and Ca using an X-ray energy of 10 keV. Many of these materials are closely related to typical low molecular organic materials, synthetic polymers, natural polymers, and biological materials such as urea, polystyrene, polymethyl methacrylate, starch, glutamic acid, stearic acid, hydroxyapatite, and so on. Data shown in Fig 1 were calculated according to Eqs (2) and (4) using *σ*_*a*_ of each element which can be obtained from a database [15]. Fig 1 demonstrates that that *μ*/*ρ* has a linear dependence on *β*/*δ* over a wide range of *β*/*δ*. Many organic materials comprising H, C, N, and O have *β*/*δ* values concentrated in the low-*β*/*δ* region (∼0.002 at 10 keV). In this region, the mass density error that results from use of the conventional approximation tends to become large because of the influence of hydrogen. From the data in Fig 1, we assumed that *μ*/*ρ* was related to *β*/*δ* through the expression

$$\begin{array}{c} (\frac{\mu }{\rho })=A(\frac{\beta }{\delta })+B, \end{array}$$ (5)

where *A* and *B* are functions of X-ray energy (*E*).

**Fig 2:**
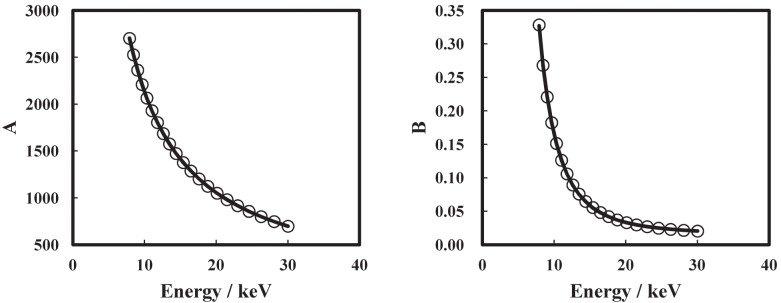
X-ray energy dependences. X-ray energy dependences of (a) *A* and (b) *B*. The solid lines are the curves of best fit.

Fig 2 shows X-ray energy dependence of the *A* and *B* between 8 keV and 30 keV. The values of *A* and *B* at each X-ray energy were obtained by weighted least-squares regression of *μ*/*ρ* as a function of *β*/*δ*. Because the values of *B* were much smaller than *μ*/*ρ* at 10 keV for the materials analyzed, as shown in Figs 1 and 2(b), *A* was assumed to be inversely proportional to *E* from Eqs (2) and (4), and the best fit curve was obtained using least-squares regression as

$$\begin{array}{c} A=2.151\times 10^{4}E^{-1}-19.27. \end{array}$$ (6)

We assumed that the X-ray energy dependence of *B* correlated strongly with the X-ray energy dependence of the *σ*_*a*_ from Eqs (2), (4) and (5). Therefore, we assumed a calibration function for *B* as

$$\begin{array}{c} B=mE^{-n}+l\sigma_{KN}, \end{array}$$ (7)

where *m*, *n*, and *l* are fitting parameters and *σ*_*KN*_ is the Klein-Nishina cross section [16]. The first term in Eq (7) corresponds to the X-ray energy dependence of the photoelectric cross section (*n* ≈ 3.1) [17]. The second term in Eq (7) is the incoherent scattering cross section. In Eq (7), X-ray energy dependence of the coherent scattering cross section is neglected because its contribution to the *σ*_*a*_ is small in the X-ray energy region investigated here (8–30 keV). The values of *m*, *n*, and *l* obtained using least-squares regression are 194.6, 3.116, and 6.534 × 10^−3^, respectively.

Using the calibration function described in Eq (5), the *μ*/*ρ* image can be calculated from the experimentally obtained *β*/*δ* image. The *μ* image can be reconstructed from the X-ray transmittance projection images using standard procedures. The *ρ* image can be calculated using the calculated *μ*/*ρ* image and the experimentally obtained *μ* image.

## Experimental

**Fig 3:**
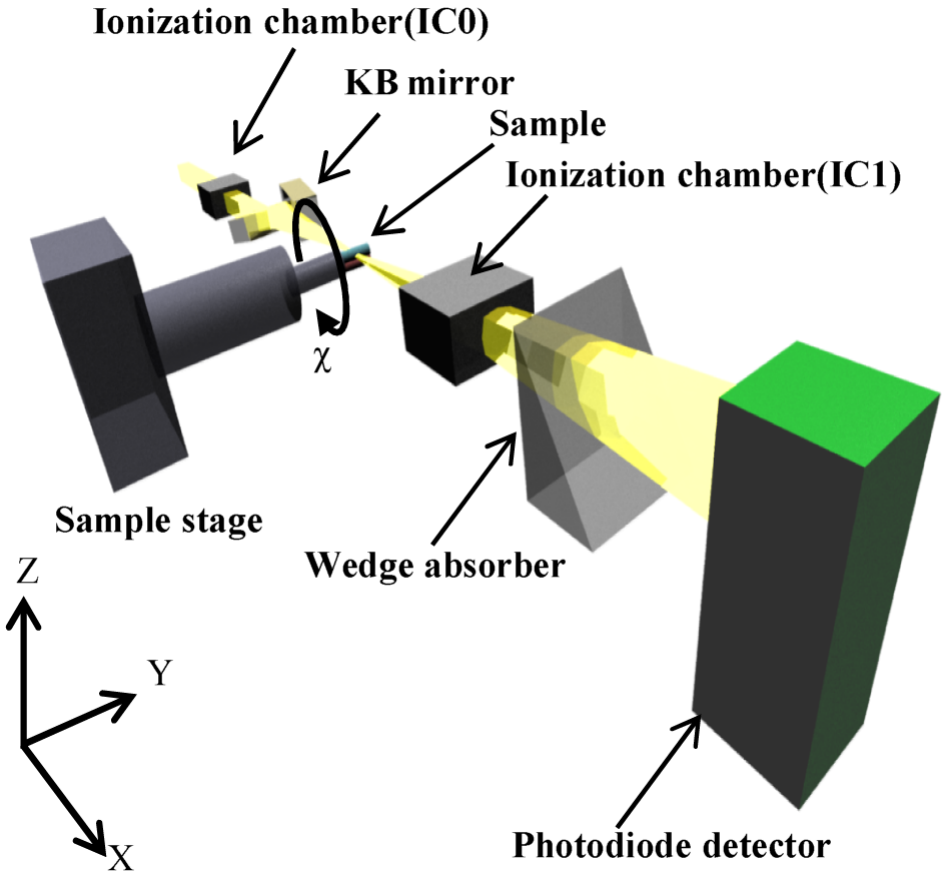
Schematic of the X-ray computed tomography system. The system comprises a KB mirror, a sample stage, two ionization chambers, a wedge absorber, and a photodiode detector. The distance from the sample to the wedge absorber is 1.5 m.

Experiments were performed using the beam line BL-4A at the Photon Factory, High Energy Accelerator Research Organization(KEK). To measure the complex X-ray refractive index for the materials studied, a scanning-type X-ray computed tomography system was developed using an X-ray microbeam and a wedge absorber. A schematic of the X-ray computed tomography system is shown in Fig 3.

Polychromatic X-rays from a bending magnet were monochromatized using a double multilayer monochromator to have an energy of 10 keV. The monochromatic X-rays were focused on the sample using a Kirkpatrick-Baez (KB) mirror [18]. The beam size was 3.5 (height) × 3.8 (width) *μ*m^2^ at the sample position. The sample was formed by gluing together fibers of polyethylene (PE) (C_2_H_4_)_*n*_ and polyethylene terephthalate (PET) (C_10_H_8_ *O*_4_)_*n*_. The sample was set on the sample stage, which consisted of X, Y, Z translation, and *χ* rotation stages, as shown in Fig 3. The X-ray transmittance was calculated using the intensities of incident and transmitted X-rays, which were detected by two ionization chambers, IC0 and IC1, placed in front of the KB mirror and behind the sample, respectively. To detect the refraction effect on the X-rays, the intensity of the X-rays transmitted through the wedge absorber was detected using an X-ray photodiode downstream from the IC1 [19, 20]. The wedge absorber was made of aluminum and the distance between the sample and the wedge absorber was 1.5 m.

The incident position of the X-rays on the wedge absorber changed when the X-rays were refracted by the sample. As a result of this displacement, the X-ray intensity detected by the photodiode changed because the path length of the X-rays in the wedge absorber changed. Therefore, the displacement of the X-rays at the wedge absorber could be calculated from the detected intensity. The refraction angle (*Δθ*) could also be calculated from the displacement and the distance between the sample and the wedge absorber. The relationship between the angular displacement of X-rays in the Z-direction and the ratio between the intensity signal detected by IC1 and the X-ray photodiode detector was established in advance by changing the position of the wedge absorber in the Z-direction without a sample present. The phase gradient for the Z-direction can be written as

$$\begin{array}{c} \frac{\partial \phi }{\partial Z}=\frac{2\pi }{\lambda }\Delta \theta . \end{array}$$ (8)

By scanning the sample in the Z-direction and rotating around the *χ*-axis, the X-ray transmittance and phase gradient at each point of the sample were obtained. The step size around the *χ*-axis was 1° and 180 projections were collected. The phase shift of the X-rays was calculated by integrating the phase gradient in the Z-direction.

## Results and Discussion

**Fig 4:**
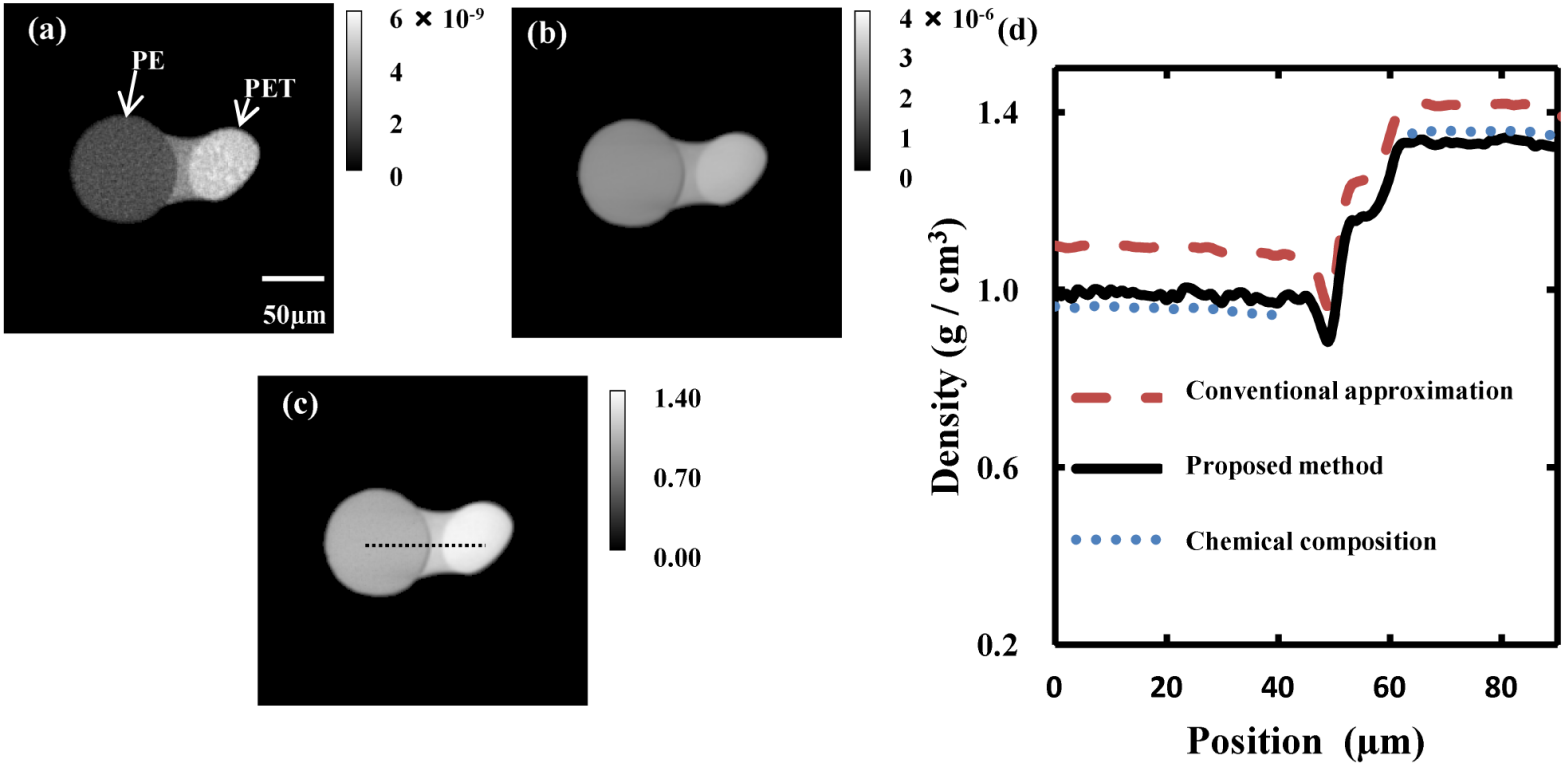
Reconstructed images. Tomographic images of (a) *β* and (b) *δ*. The *β* image is linearly proportional to the *μ* image as shown in Eq (2). (c) Mass density image in g/cm^3^, (d) line profiles of mass density along the broken line in (c). The black solid, red dashed, and blue dotted lines show the mass density calculated by the proposed method, conventional approximation, and Eq (3) using a known chemical composition, respectively.

The reconstructed distributions of *β* and *δ* calculated from the tomographic images are shown in Fig 4(a) and 4(b), respectively. These images were reconstructed using the algebraic reconstruction technique (ART) [21] with 100 iterations. In Fig 4(a) and 4(b), the contrast difference between the two polymer fibers can be easily discriminated by eye. The right and left polymer fibers were PET and PE, respectively. It is also possible to see the glue that connects the two polymer fibers from the images in Fig 4(a) and 4(b). Fig 4(c) presents a tomographic *ρ* image, which was calculated using the reconstructed *β* and *δ* images (Fig 4(a) and 4(b), respectively) and a calibration function (Eq (3)). The profile along the broken line indicated on the *ρ* image is plotted with a black solid line in Fig 4(d). The red dashed line in Fig 4(d) is the *ρ* value calculated using the conventional approximation, which assumes that ⟨*Z* + *f*′⟩/⟨*A*⟩ is equal to 0.5. The blue dotted lines in Fig 4(d) show the mass density of PE (left) and PET (right) calculated from the *δ* image using Eq (3) with the known chemical composition. The mass densities of PE and PET obtained were 0.95 ± 0.01 g/cm^3^ and 1.34 ± 0.02 g/cm^3^, respectively. This result agrees well with the tabulated value for these polymers (PE: 0.910–0.965 g/cm^3^, PET: 1.33–1.42 g/cm^3^) [22]. The mass densities for PE and PET obtained using the proposed method (black solid line in Fig 4(d)) are in agreement with the calculated values (blue dotted lines in Fig 4(d)), while the conventional approximation (red dashed line in Fig 4(d)) shows a large discrepancy in both cases. The average difference in the mass density measured with the present method and the calculated mass density is approximately 2% for PET and 3% for PE. As expected, the large hydrogen content in these materials results in a large error using the conventional approximation (the hydrogen content, based on the number of atoms, of PET and PE are 36% and 67%, respectively).

Having successfully evaluated the mass density of organic materials having an unknown composition, the proposed method could be used for nondestructive characterization of other materials, in combination with the electron density obtained from *δ* tomography [11] and the effective atomic number obtained from absorption and phase contrast imaging [23]. Moreover, this method could be applied to other X-ray imaging techniques that simultaneously acquire quantitative information about *β* and *δ*, such as scanning phase-contrast X-ray computed tomography [24, 25] and X-ray phase computed tomography with a grating interferometer [26]. Although we have only experimentally demonstrated this method using materials comprising H, C, N, and O (PET and PE), the strong relationship between *μ*/*ρ* and *β*/*δ* has also been demonstrated for materials comprising heavier elements, such as S, P, and Ca, at X-ray energies between 8 keV and 30 keV, as shown in Figs 1 and 2. Therefore, this method could be effectively used for many organic materials, including biopolymers.

## Conclusions

The distribution of the complex X-ray refractive index for the polymer fibers was obtained from a scanning-type X-ray tomography system using an X-ray microbeam and wedge absorber. The *β*/*δ* has a strong relationship with the mass attenuation coefficient of organic materials comprising H, C, N, O, Na, Mg, P, S, Cl, K, and Ca at X-ray energies ranging from 8 keV to 30 keV. Using this relationship, the mass density image of low-Z materials having unknown chemical compositions can be obtained from the absorption and phase contrast images. The present method is superior to the conventional method, which assumes that ⟨*Z* + *f*′⟩/⟨*A*⟩ is equal to 0.5, especially for materials with a large hydrogen content (i.e., low *β*/*δ* materials). This technique will be applied to a wide range of materials in future studies.
